# Anesthesia and Airway Management in a Child with Frank Ter Haar Syndrome Suspected Difficult Airway Undergoing Cardiac Surgery: A Case Report

**DOI:** 10.5812/aapm-144682

**Published:** 2024-09-14

**Authors:** Maryam Ghadimi, Yasmin Chaibakhsh, Mohsen Ziyaeifard

**Affiliations:** 1 Rajaie Cardiovascular Medical and Research Center, Iran University of Medical Sciences, Tehran, Iran

**Keywords:** Frank-ter Haar, Difficult Airway, Cardiac Surgery

## Abstract

**Introduction:**

Frank ter Haar syndrome (FTHS) is a rare and complex multisystem congenital genetic disorder that leads to craniofacial, cardiac, and skeletal abnormalities. We report the anesthesia and airway management of a child with FTHS who was referred for repair of atrial septal defect (ASD) and ventricular septal defect (VSD).

**Case Presentation:**

The patient exhibited craniofacial and skeletal abnormalities, including craniosynostosis, micrognathia, a prominent forehead, hypertelorism, and anteverted nostrils. These features raised the possibility of a difficult airway.

**Conclusions:**

For patients with potential difficult airways undergoing elective surgery, the procedure should be postponed until all necessary equipment for managing a difficult airway is available.

## 1. Introduction

The first case of Frank ter Haar syndrome (FTHS) was reported in 1973 by Frank et al., who identified megalocornea and several skeletal anomalies. Later, Ter-Haar et al. described a similar presentation in a family. A homozygous mutation in the SH3PXD2B gene has been identified as the cause of FTHS, primarily in children with consanguineous parents, though non-consanguineous cases have also been reported (1). Podosome and invadopodia adhesion types play a crucial role in remodeling and motility of the extracellular matrix (ECM) by forming finger-like protrusions that interact with matrix metalloproteinases in the surrounding ECM (2-4).

Frank ter Haar syndrome is characterized by brachycephaly, wide fontanels, frontal prominence, glaucoma, megalocornea, micrognathia, hypertelorism, skeletal abnormalities and deformities, as well as cardiac defects such as atrial septal defect (ASD), ventricular septal defect (VSD), and mitral valve deformity (5-7). Dental abnormalities, respiratory underdevelopment, and retinal detachment have also been reported in some cases (8, 9). Given that survival in FTHS is closely linked to the timely management of serious anomalies, such as cardiac or respiratory issues, we present a case of FTHS with ASD and VSD, along with its surgical and anesthetic management.

## 2. Case Presentation

After obtaining the ethics approval number IR.RHC.REC.1402.032 from our institute, we reported this case.

A 3-year-old male child, weighing 14 kilograms and measuring 95 centimeters in height, was referred for the repair of his ASD and VSD. The medical history revealed several flu-like episodes over the past years, along with recurrent cough and occasional snoring. The patient had previously undergone surgery for undescended testicles a year and a half ago. His father also reported ASD and VSD but had no history of surgical correction. Both parents had a history of asthma.

The ASD and VSD were diagnosed by a cardiologist shortly after birth, and appropriate medication was initiated. Upon arrival at our facility, the patient’s rough skin and coarse wrinkles prompted further evaluation for any underlying syndromic condition. The patient was subsequently diagnosed with FTH syndrome and was referred to our hospital for repair of the ASD and VSD.

**Figure 1.**
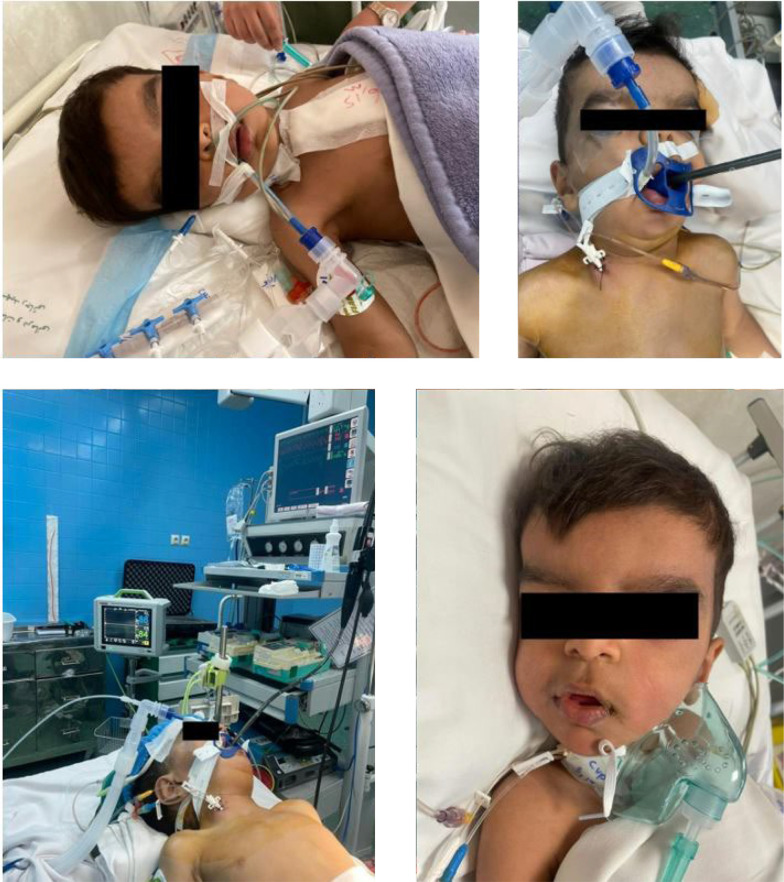
A 3-year-old male child with Frank-ter Haar Syndrome

Preoperative evaluations included neurologic, pulmonary, and endocrinologic assessments. Clinical examination revealed craniosynostosis, micrognathia, a prominent forehead, hypertelorism, and anteverted nostrils (Figure 1). The patient had a relatively large trunk and thin arms and legs. Neurologic and endocrinologic evaluations were unremarkable.

The pulmonary evaluation showed only mild tachypnea. A complete cardiac workup revealed a left ventricular ejection fraction of 55%, moderate tricuspid regurgitation, mild to moderate mitral regurgitation, a cleft in the mitral valve, mild pulmonic insufficiency, pulmonary valve stenosis, and a pulmonary artery pressure of 25 mmHg. No aortic insufficiency or aortic stenosis was reported. The sizes of the first and second ASDs were reported as 0.9 cm and 0.65 cm, respectively. Angiography showed a cleft in the anterior mitral leaflet, moderate mitral regurgitation, bilateral atrial enlargement, a normal left ventricle, and right ventricular enlargement. Computed tomography angiography revealed a primum ASD, a small inlet-type VSD, a small peri-membranous VSD, a dilated main pulmonary artery, and normal coronary artery origin and course. Laboratory tests were normal.

After obtaining informed consent from the parents, the patient was prepared for surgery. Anticipating difficult and complex intubation, all necessary equipment, including bougies, laryngeal masks in all sizes, tracheal tubes in all sizes, and a pediatric fiberoptic bronchoscope, were prepared. The patient was transferred to the operating room with a size-22 peripheral intravenous line. Pulse oximetry, electrocardiogram, and non-invasive blood pressure monitoring were set up. An oxygen mask was used for proper ventilation, and anesthesia induction was achieved with sevoflurane. Following laryngoscopy and visualization of the vocal cords and epiglottis, intubation was successfully performed. The patient then received midazolam (0.2 µg/kg), fentanyl (3 µg/kg), and rocuronium (0.6 mg/kg). Intubation was completed with a 4.5-size, cuffless endotracheal tube. After confirming bilateral lung ventilation, the patient was connected to a mechanical ventilator (tidal volume: 140 cc, respiratory rate: 20/minute, PEEP: 2 cm H_2_O). Following prepping and draping, the left brachial artery was cannulated, and an arterial blood sample was taken. For central venous line access, the right internal jugular vein was catheterized. Anesthesia was maintained with midazolam (1 µg/kg/min), fentanyl (5 µg/kg/hr), and cisatracurium (1 µg/kg/min). Bilateral cerebral oximetry was used to monitor cerebral oxygen saturation, and a transesophageal echocardiographic probe was used to assess for any cardiac complications. Urinary catheterization was performed to monitor urinary output.

The operation began after positioning the patient. After sternotomy and thymectomy, arterial and venous cannulation were performed, followed by the initiation of cardiopulmonary bypass. The VSD, ASD1, ASD2, and mitral valves were then repaired. Throughout the surgery, cerebral oxygen saturation remained within the normal range, and urinary output was normal. Cardiopulmonary support was discontinued with the administration of epinephrine (0.1 µg/kg/min) and milrinone (0.5 µg/kg/min). A pediatric echocardiologist assessed cardiac function via transesophageal echocardiography. After closing the sternum and completing suturing, the patient was transferred from the operating room to the pediatric cardiac intensive care unit (PCICU) with stable hemodynamics. Mechanical ventilation was maintained upon admission to the PCICU. Following recovery from anesthesia, sedation was continued with fentanyl (1 - 2 µg/kg/hr). After 24 hours, and after confirming the stability of the patient’s condition, absence of drainage, normal urinary output, normal blood gases, and normal consciousness, weaning from the mechanical ventilator was performed, and the patient was extubated.

Due to a type 2 cardiac block, the patient was monitored in the PCICU for 5 days. The patient was then discharged from the PCICU and transferred to the surgical ward. After 48 hours, the patient was discharged from the hospital with normal sinus rhythm, normal laboratory tests, normal vital signs, and stable condition. Follow-up procedures and intervals were thoroughly explained to the parents.

## 3. Discussion

Frank Ter Haar syndrome is a rare multisystem genetic condition characterized by significant skeletal, cardiac, and craniofacial deformities. It is caused by an autosomal recessive mutation in the SH3PXD2B gene, which is involved in ECM remodeling and cell migration (10). The primary manifestations of FTHS include hypertelorism, bulging eyes, a prominent coccyx, a prominent forehead, brachycephaly, and macrocornea, with or without glaucoma. Facial abnormalities such as a small chin, full cheeks, and deformed fingers are also common (11). Atrial and VSDs are frequently reported in cases of FTHS. Our patient exhibited craniosynostosis, micrognathia, a prominent forehead, hypertelorism, and anteverted nostrils, all of which are typical findings in FTHS cases.

To date, more than 40 cases of FTHS have been reported. Primarily, macrocornea, glaucoma, hypertelorism, and colobomas have been identified as ocular and orbital abnormalities associated with FTHS (12). However, recent cases have reported retinal detachment as a novel manifestation of this syndrome (8).

In 2012, Iqbal et al. published a comprehensive study investigating the causes and genetic factors involved in FTHS. They conducted genetic mapping on 16 cases of FTHS from 12 unrelated consanguineous families. The primary hypothesis was that a homozygous mutation in SH3PXD2B could cause FTHS. By analyzing TKS4 levels in fibroblasts from these cases, they found that a loss-of-function mutation in SH3PXD2B was likely the primary cause of FTHS, as normal intracellular levels of SH3PXD2B transcript were present. However, no mutation was detected in six cases. Further analysis revealed that, although normal mRNA levels of SH3PXD2B were detected, TKS4 was barely present in the fibroblasts. This was attributed to undetected mutations affecting TKS4 stability or synthesis. Mutations in other genes responsible for TKS4 regulation were also observed in the same patients. Therefore, it is evident that FTHS cannot be solely attributed to SH3PXD2B mutations. This should be considered during the diagnostic process, especially when suspected cases of FTHS test negative for SH3PXD2B mutations (1).

Difficult intubation in children with a history of heart disease and syndromic conditions should always be anticipated (9).

The surgical and anesthetic management of FTHS is always challenging due to the apparent craniofacial and cardiac abnormalities. In this case, the patient received fentanyl and midazolam for anesthesia, which are known to have prolonged effects and short clinical manifestations when administered continuously (13). Basaran et al. (7) reported a similar case involving atrial and ventricular septal defects (ASD/VSD) and severe kyphoscoliosis. Their patient also experienced repeated episodes of fever, coughing, and respiratory infections, akin to our case. These recurrent infections may be due to compromised immune systems; a factor that has not been thoroughly studied in the current literature. Another contributing factor could be the underdevelopment of the nasomaxillary complex and the pattern of mouth breathing in these patients. Additionally, they used intravenous anesthesia with fentanyl (1 mcg/kg) and rocuronium (0.6 mg/kg). These issues should be considered in future cases.

Unlike our case, their attempt at intubation using a video laryngoscope failed due to the complex access resulting from severe kyphoscoliosis. Their second attempt, using a laryngeal mask airway, was successful for intubation and ventilation. In another recent case, Tommasino and Albicini used video laryngoscopy and a fiberoptic bronchoscope for intubation. In both cases, due to suspected complex intubation, the necessary equipment was prepared in advance (7, 14). There are few case reports regarding ASD/VSD and kyphoscoliosis in FTHS, and the anesthesia management for such surgeries is challenging due to potential airway complications, such as gastroesophageal reflux, bronchopulmonary hypoplasia, and alterations in respiratory drive (15). Therefore, certain considerations should be taken before any operation, including checking airway intubation or ventilation before administering blocking medications and ensuring adequate bleeding control (7).

### 3.1. Conclusions

Given the higher likelihood of difficult airway management in patients with syndromic disorders and congenital heart diseases—due to factors such as a small jaw, large tongue, and improper positioning of teeth—preoperative anesthesia evaluation should be conducted with increased caution. For patients with a potentially difficult airway undergoing elective surgery, the procedure should be postponed until all necessary equipment for managing a difficult airway is available. In the current case, fentanyl, midazolam, and cisatracurium were used for anesthesia, and intubation was performed after confirming the proper function of the vocal cords and epiglottis using laryngoscopy. However, due to the airway abnormalities associated with FTHS, thorough examination of the epiglottis and vocal cords is strongly recommended to ensure effective maintenance of anesthesia.

## Data Availability

The dataset presented in the study is available on request from the corresponding author during submission or after publication.
